# Developmental and Light-Induced Expression Patterns of Phototransduction Pathway Genes in Large Yellow Croaker (*Larimichthys crocea*)

**DOI:** 10.3390/genes17070788

**Published:** 2026-07-10

**Authors:** Shaocong Huang, Hao Xu, Weiqing Huang, Lizhen Li, Qun Ji, Tongtong Wu, Longhao Wu, Wei Song

**Affiliations:** 1 East China Sea Fisheries Research Institute, Chinese Academy of Fishery Sciences, Shanghai 200090, China; 2 College of Fisheries and Life Science, Shanghai Ocean University, Shanghai 201306, China; 3 Qingdao Marine Science and Technology Center, Qingdao 266200, China; 4 College of Marine Sciences, Ningde Normal University, Ningde 352100, China; 5 Ningde Dingcheng Fishery Company Limited, Ningde 352100, China; 6 National Engineering Research Center for Marine Aquaculture, Zhejiang Ocean University, Zhoushan 316022, China

**Keywords:** *Larimichthys crocea*, phototransduction pathway, bioinformatics, visual development

## Abstract

Background: The visual system plays critical roles in fish feeding, predator avoidance, and reproduction, with the phototransduction pathway serving as the core molecular mechanism of visual signal transduction. This study represents the first systematic analysis of phototransduction-related genes in large yellow croaker (*Larimichthys crocea*). Methods: Bioinformatics, transcriptomic analysis, and RT-qPCR were used to identify phototransduction-related genes in large yellow croaker and analyze their developmental expression patterns and responses to different light spectra. Results: Thirty phototransduction-related genes were identified, including genes involved in light perception, signal amplification, and signal termination and recovery. Structural analyses indicated that these genes were highly conserved in teleosts. Most genes showed low expression during 1–7 days post-hatching (dph), increased expression during 7–24 dph, and stable expression during 24–35 dph. Collectively, these results identify 15–24 dph as a critical developmental window for visual system maturation in large yellow croaker larvae. Under different spectral treatments at 15 dph, phototransduction-related genes exhibited wavelength-dependent expression patterns. Under the present experimental conditions, relatively higher expression levels of most phototransduction-related genes were observed under blue- and green-light treatments, whereas relatively lower expression levels were observed under the red-light treatment. Key downstream genes, including *gucy2f*, *guca1d*, *calml4a*, and *rgs9b*, were significantly affected by different light spectra. Conclusions: This study revealed the developmental dynamics and spectral response characteristics of phototransduction-related genes in the large yellow croaker. These findings provide new insights into teleost visual development and light adaptation and provide a molecular basis for future studies aimed at optimizing light conditions during larval rearing in large yellow croaker aquaculture.

## 1. Introduction

Vision is a critical sensory system in fish, playing essential roles in feeding, predator avoidance, habitat selection, and reproduction [1]. During the larval and juvenile stages, visual system development directly determines first feeding, prey recognition, and early survival [2]. Light is an important environmental factor that influences retinal development [3], photoreceptor differentiation, visual sensitivity [4], and the expression of vision-related genes, thereby regulating feeding behavior, circadian rhythms, stress responses, and environmental adaptation [5]. Fish adapt to their respective light environments through the regulation of vision-related gene expression; consequently, the light environment is considered a key ecological factor influencing larval growth, development, and survival [6]. Therefore, elucidating the molecular mechanisms underlying light-regulated visual development is essential for understanding the adaptive strategies of fish [7].

Large yellow croaker (*Larimichthys crocea*) belongs to the family Sciaenidae of the order Perciformes [8] and primarily inhabits shallow coastal waters. These habitats are characterized by dynamic light conditions resulting from variations in water depth, turbidity, weather, and diel cycles. Consequently, proper visual system development and adaptation to changing light environments are essential for feeding, habitat utilization, and survival during the early life stages of this species. Large yellow croaker is one of the most economically important marine aquaculture species in China, with an aquaculture production of 292,600 tons in 2024 [9]. However, larval rearing practices and seed production techniques still require further optimization. Previous studies on large yellow croaker have mainly focused on nutritional regulation [10], growth performance [11,12,13], and responses to environmental stress [14,15]. In contrast, studies on visual development remain limited. To date, available studies have mainly characterized the opsin gene repertoire and examined the expression patterns of opsin genes under different light spectra [16,17,18,19,20,21]. Phototransduction, the major downstream signaling pathway of opsins, converts light stimuli into neural signals and plays a central role in visual signal transduction [22]. In model fish species such as zebrafish (*Danio rerio*), several phototransduction-related genes have been reported to exhibit distinct developmental expression patterns and differential responses to changes in light conditions (Figure S1) [23]. However, these studies have been largely confined to model fish species, and information regarding marine aquaculture species, including large yellow croaker, remains limited [24]. In particular, the composition of phototransduction-related genes, their developmental expression dynamics during larval and juvenile stages, and their transcriptional responses to different light spectra have not been systematically investigated. These gaps in knowledge limit our understanding of visual development and light adaptation in large yellow croaker and hinder the application of spectral regulation strategies in larval rearing [25]. Therefore, systematic characterization of phototransduction-related genes and their developmental and spectral expression patterns is essential for elucidating the molecular mechanisms underlying visual system development in this species [26].

The present study systematically identified and characterized phototransduction-related genes in large yellow croaker using genome and transcriptome datasets. We further employed real-time quantitative PCR (RT-qPCR) to examine their expression profiles across developmental stages and under different light spectral conditions. Given that artificial hatchery environments differ substantially from natural light environments, while visual development directly affects first feeding and prey recognition in larvae, the widespread use of continuous white-light illumination in large yellow croaker hatcheries warrants further evaluation. We hypothesized that phototransduction-related genes exhibit stage-specific expression patterns during early development and respond differentially to distinct light spectra, thereby contributing to visual system maturation and adaptation to changing light environments. The findings of this study provide new insights into the molecular basis of visual development and light adaptation in teleosts and provide a theoretical foundation for optimizing light management strategies and improving larval rearing practices in large yellow croaker aquaculture.

## 2. Materials and Methods

### 2.1. Ethics Statement

All experimental procedures were conducted in accordance with the guidelines of the Chinese Academy of Sciences (IACUC approval #160413; approval date: 1 March 2024). This study did not involve endangered or protected species. Prior to sample collection, all large yellow croaker were euthanized by an overdose of eugenol (Beyotime, Shanghai, China) (200 mg/L) to minimize animal suffering. Death was confirmed by the absence of response to tactile stimulation before sample collection.

### 2.2. Experimental Fish and Sampling Time Points

Larval and juvenile *L. crocea* used in this study were obtained from a commercial hatchery in Ningde, Fujian Province, China. Fish were reared under aquaculture conditions at 24.5–25.5 °C from hatching. Behavioral observations were conducted daily at 9:00 a.m. for 40 consecutive days after hatching to monitor the development of visual responses. Five representative developmental stages, including 1, 7, 15, 24, and 35 days post-hatching (dph), were selected for subsequent analyses. All samples were collected at approximately 10:00 a.m. on each sampling day to minimize potential effects of diurnal variation on gene expression.

The 15 dph larvae of *L. crocea* were randomly assigned to four spectral treatments: white, blue (peak wavelength: 449 nm), green (516 nm), and red light (635 nm), with three biological replicates per treatment. All light treatments were provided using identical LED lamps under the same installation height and illumination conditions. The emission spectra of the LED light sources were measured using a spectroradiometer (Konica Minolta CS-2000, Konica Minolta, Tokyo, Japan), confirming that each light source exhibited a single dominant wavelength without secondary spectral peaks and represented the red, green, and blue regions of the visible spectrum. The irradiance and photon flux density of the different spectral treatments were not quantified or standardized in the present study.

Each treatment consisted of one tank containing three identical culture containers, with LED lamps positioned centrally above the tanks. Larvae were exposed to the experimental light conditions for 72 h. During the experiment, water temperature was maintained at 24 °C using a thermostatic water bath system, continuous aeration was provided, and 50% of the water was renewed daily. Tanks were covered with blackout cloth to prevent external light interference, and the photoperiod was set at 24 L:0 D. Samples were collected immediately after the exposure period.

**Figure 1:**
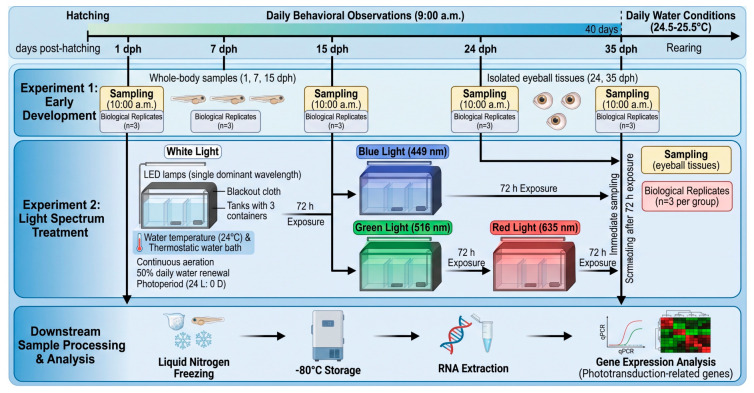
Schematic overview of the experimental design and workflow.

To investigate the expression patterns of phototransduction-related genes during early development and under different light spectrum treatments, samples were collected for subsequent RNA extraction and gene expression analysis. Whole-body samples were collected at 1, 7, and 15 dph, whereas isolated eyeball tissues were collected at 24 and 35 dph because the eyes could be accurately dissected at these stages. For the light spectrum experiment, eyeball tissues were collected from fish in each spectral treatment group for subsequent RNA extraction and gene expression analysis. All samples were immediately frozen in liquid nitrogen and stored at −80 °C until further analysis. Three biological replicates were collected for each experimental group and processed under identical conditions (Figure 1).

### 2.3. RNA Extraction and cDNA Synthesis

Eye tissues from three individuals per group were collected at five developmental stages. Total RNA was extracted using TRIzol reagent (Invitrogen, Carlsbad, CA, USA) following the manufacturer’s instructions. mRNA was subsequently purified and fragmented for cDNA library construction using the Illumina mRNA-Seq library preparation kit (Illumina, San Diego, CA, USA).

For spectral experiment samples, total RNA was extracted using the RNA Easy Fast Animal Tissue/Cell Total RNA Extraction Kit (Tiangen, Beijing, China). RNA quality and concentration were assessed using a NanoDrop OneC spectrophotometer (Thermo Fisher Scientific, Waltham, MA, USA), and RNA integrity was verified by 1% agarose gel electrophoresis. cDNA was synthesized using the FastKing One-Step gDNA Removal and cDNA First-Strand Synthesis SuperMix kit (Tiangen, Beijing, China) for RT-qPCR analysis.

### 2.4. Transcriptome Sequencing and Analysis

Sequencing was performed by Shanghai Yingbai Biotechnology Co., Ltd. (Shanghai, China). Paired-end sequencing (2 × 100 bp) was conducted on the Illumina HiSeq 2000 platform (Illumina, San Diego, CA, USA) with an average insert size of 300 ± 50 bp. Raw FASTQ files were first assessed for sequencing quality using FastQC version 0.12.1. Adapter sequences and low-quality bases were removed using Cutadapt version 4.9 to generate clean reads. The clean reads were subsequently aligned to the *L. crocea* reference genome using HISAT2 version 2.2.1. Gene expression levels were quantified as fragments per kilobase of transcript per million mapped reads (FPKM). The expression levels (FPKM) of phototransduction pathway-related genes in large yellow croaker were obtained from the transcriptome sequencing data. Three pooled eye tissue samples were collected from each of five developmental stages (1, 7, 15, 24, and 35 dph). Differentially expressed genes (DEGs) were identified using DESeq2 version 1.42.1 with thresholds of |log_2_ fold change| > 1 and FDR < 0.05. Gene Ontology (GO) and Kyoto Encyclopedia of Genes and Genomes (KEGG) enrichment analyses were conducted using clusterProfiler version 4.10.1, with adjusted *p* < 0.05 considered significant.

### 2.5. Genome-Wide Screening, Identification, and Evolutionary Analysis of Phototransduction Pathway-Related Genes

Phototransduction pathway-related gene sequences were initially identified from the large yellow croaker transcriptome database constructed in our laboratory. Candidate genes were first annotated based on KEGG pathway mapping and GO functional enrichment related to the phototransduction pathway. Only transcripts consistently annotated as phototransduction pathway-related genes in both KEGG and GO databases were retained as candidate genes. The large yellow croaker genome sequence and annotation files (GCF_000972845.2) were downloaded from the NCBI database to further confirm gene identity. Candidate transcript sequences were aligned to the reference genome to verify gene loci and annotation consistency.

ORF-Finder (https://www.ncbi.nlm.nih.gov/orffinder/, accessed on 1 January 2026) was utilized to predict the encoded amino acid sequences of phototransduction pathway-related genes (Table S1). Conserved domains were identified using the Conserved Domain tool to support functional classification of phototransduction pathway-related proteins (https://www.ncbi.nlm.nih.gov/Structure/bwrpsb/bwrpsb.cgi, accessed on 1 January 2026). TBtools v2.423 was employed to visualize chromosomal locations and conserved domains of phototransduction pathway-related genes. Physicochemical properties were analyzed using the ExPASy server (https://www.expasy.org/, accessed on 3 January 2026). Subcellular localization of phototransduction pathway-related proteins was predicted via Cell-PLoc 2.0 (http://www.csbio.sjtu.edu.cn/bioinf/Cell-PLoc-2/, accessed on 5 January 2026). Protein domain features were analyzed using SMART (https://smart.embl.de/, accessed on 15 January 2026). Secondary structures of phototransduction pathway-related proteins were predicted through SOPMA (https://web.expasy.org/protscale/, accessed on 5 January 2026), and three-dimensional protein structure models were constructed using SWISS-MODEL (https://swissmodel.expasy.org/, accessed on 5 January 2026). Schematic diagrams were generated using BioGDP (https://www.biogdp.com, accessed on 15 January 2026).

### 2.6. RT-qPCR Analysis

Gene expression levels were quantified by RT-qPCR using the SYBR Green method, with *β-actin* and *18S rRNA* serving as reference genes. Primer sequences are provided in the Supplementary Materials (Table S2). Relative gene expression levels were calculated using the 2^−ΔΔCt^ method. Three independent biological replicates were analyzed for each experimental group. Each biological replicate consisted of pooled tissues from three fish, from which RNA was independently extracted and reverse-transcribed into cDNA. For each biological replicate, the same cDNA sample was subjected to three independent PCR amplifications as technical replicates. Primer specificity was confirmed by melting curve analysis.

The RT-qPCR reaction mixture (20 μL total volume) consisted of 10 μL 2 × FastReal qPCR premix (SYBR Green, Tiangen, Beijing, China), 0.6 μL each of forward and reverse primers, 7.8 μL ddH_2_O, and 1 μL cDNA template. Thermal cycling conditions were 95 °C for 2 min (initial denaturation), followed by 40 cycles of 95 °C for 5 s (denaturation), 52 °C for 10 s (annealing), and 72 °C for 15 s (extension).

### 2.7. Statistical Analysis

Data were statistically analyzed using SPSS 26.0 software. All data were derived from three independent biological replicates and are expressed as mean ± standard deviation. One-way analysis of variance (ANOVA) was used for intergroup comparisons. Model residuals were subsequently evaluated for normality using the Shapiro–Wilk test, and homogeneity of variance was assessed using Levene’s test to verify the ANOVA assumptions. Given the limited number of biological replicates (*n* = 3), the results of the normality test were interpreted with caution. When the assumptions were satisfied, pairwise comparisons were performed using Tukey’s honestly significant difference (HSD) test. Different letters indicate significant differences, with the significance level set at *p* < 0.05. The consistency between RNA-seq and RT-qPCR results was evaluated using Spearman’s rank correlation analysis based on the expression values of the selected genes, with *p* < 0.05 considered statistically significant. Graphs were generated using GraphPad Prism 9.0 software.

## 3. Results

### 3.1. Chromosomal Distribution of Phototransduction Pathway-Related Genes in Large Yellow Croaker

**Figure 2:**
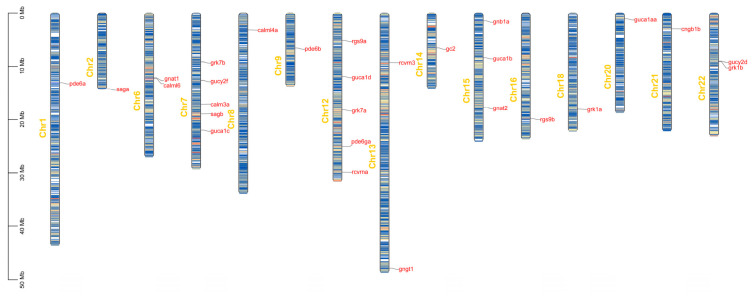
Chromosomal distribution of phototransduction-related genes in *Larimichthys crocea*. The left scale indicates chromosome length in megabase pairs (Mb). The right panel shows the chromosomal distribution and genomic positions of phototransduction-related genes across each chromosome.

A set of phototransduction pathway-related genes was identified in the large yellow croaker genome, and their chromosomal distribution patterns were systematically characterized. Results revealed that these genes were distributed across chromosomes 1, 2, 6, 7, 8, 9, 12, 13, 14, 15, 16, 18, 20, 21, and 22 (Figure 2), indicating that phototransduction-related genes are distributed across multiple chromosomes in the genome.

### 3.2. Physicochemical Properties and Subcellular Localization Prediction of Phototransduction Pathway-Related Genes in Large Yellow Croaker

The basic physicochemical properties of the proteins encoded by the identified phototransduction-related genes were analyzed. The predicted protein lengths, molecular weights, and theoretical isoelectric points varied across different gene families (Table S3). Subcellular localization prediction showed that most proteins were predicted to localize to the cell membrane or cytoplasm, while a subset of proteins was additionally predicted to localize to other cellular compartments, including the nucleus, Golgi apparatus, extracellular region, and mitochondria.

### 3.3. Functional Modular Classification of Phototransduction Genes

Based on GO MF enrichment analysis, the identified phototransduction-related genes were classified into four functional modules (Table S4). The photon capture and activation module includes the opsin genes *rho* and *rh2a*. The signal amplification module comprises G protein subunits and phosphodiesterases, including *gnat1*, *gnat2*, *pde6a*, *pde6b*, *pde6ga*, *gngt1*, and *gnb1a*. The ion flux and electrophysiological response module includes *cngb1b*, *calm3a*, *calml4a*, *calml6*, *guca1a*, *guca1b*, *guca1c*, and *guca1d*. The termination and recovery module consists of *gucy2d*, *gucy2f*, *gc2*, *grk1a*, *grk1b*, *grk7a*, *grk7b*, *sagb*, *saga*, *rgs9a*, *rgs9b*, *rcvrna*, and *rcvrn3*.

### 3.4. Conserved Domain Analysis

**Figure 3:**
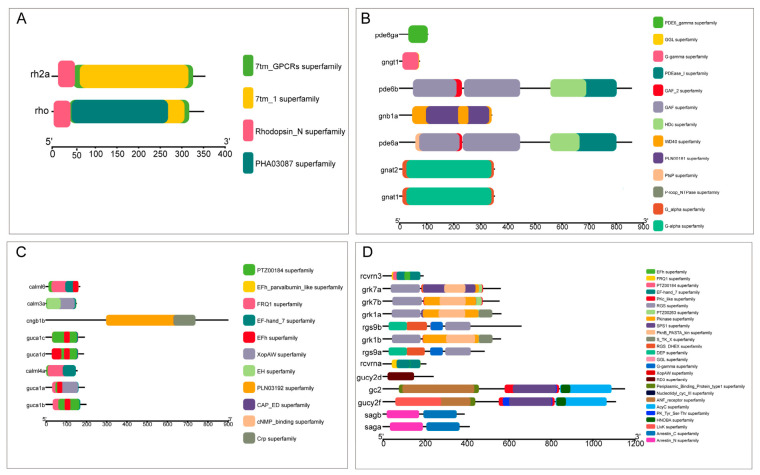
Conserved domain architecture of phototransduction-related proteins in *L. crocea*: (**A**) Domain organization of photoreception proteins (rho and rh2a). (**B**) Core signal transduction components, including G proteins and phosphodiesterases. (**C**) Calcium signaling and channel regulation proteins. (**D**) Signal termination and recovery proteins. Different colors represent different conserved protein domains. Some conserved domains overlap extensively within the same amino acid regions. Consequently, domains with shorter spans may be completely masked by larger overlapping domains in the graphical representation, although they are included in the domain annotation.

Conserved domain analysis demonstrated that phototransduction pathway-related proteins possessed the characteristic domains required for their respective functions along the phototransduction cascade (Figure 3). At the photoreception stage, rho and rh2a contained the conserved rhodopsin N-terminal domain and seven-transmembrane GPCR domain (Figure 3A). Core signal transduction proteins, including G proteins and phosphodiesterases, harbored the canonical G protein-related, WD40, GAF, and PDEase domains (Figure 3B). Calcium signaling and channel regulation proteins contained conserved EF-hand and cyclic nucleotide-binding domains (Figure 3C), whereas proteins involved in signal termination and recovery possessed characteristic RGS, kinase, and arrestin domains (Figure 3D). Overall, the conserved domain organization was highly consistent with the predicted functional roles of these proteins in the phototransduction pathway.

### 3.5. Secondary and Tertiary Structure Prediction of Phototransduction-Related Proteins

**Figure 4:**
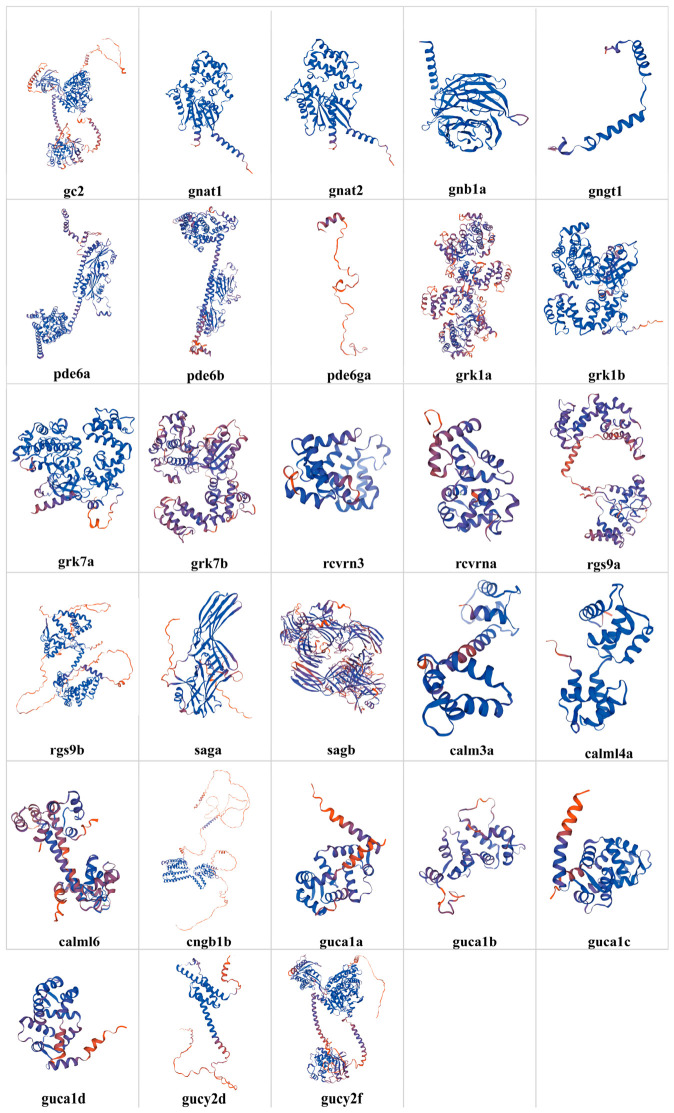
Three-dimensional structure prediction of phototransduction-related proteins in *L. crocea*.

Secondary structure prediction showed that phototransduction-related proteins displayed similar overall structural compositions, with α-helices and random coils representing the predominant secondary structural elements, whereas β-sheets accounted for a relatively smaller proportion (Table S3). Based on these predictions, three-dimensional structural models were generated for all phototransduction-related proteins. The predicted tertiary structures were primarily composed of α-helices, random coils, and extended strands (Figure 4), which were generally consistent with the secondary structure predictions.

### 3.6. Differential Expression Analysis of Phototransduction-Related Genes in L. crocea

**Figure 5:**
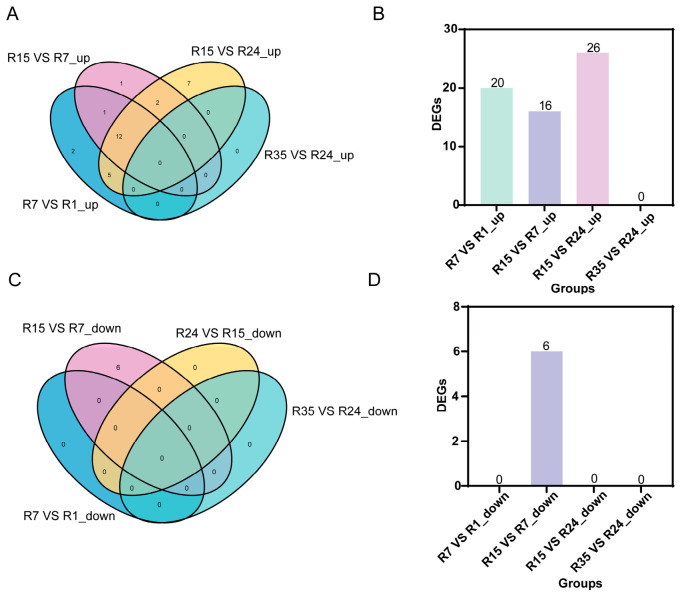
Number of differentially expressed phototransduction-related genes during early developmental stages of *L. crocea*: (**A**) Venn diagram of upregulated differentially expressed genes. (**B**) Bar chart showing the number of upregulated differentially expressed genes. (**C**) Venn diagram of downregulated differentially expressed genes. (**D**) Bar chart showing the number of downregulated differentially expressed genes. RNA-seq analysis was performed using three independent biological replicates for each developmental stage (*n* = 3).

Venn diagram analysis (Figure 5) comparing the distribution characteristics of differentially expressed genes between consecutive developmental stages revealed distinct stage-specific changes in phototransduction gene expression.

During the 1–7 dph stage, 20 phototransduction-related genes were significantly upregulated; during the 7–15 dph stage, 16 genes remained upregulated; during the 15–24 dph stage, the number of upregulated genes further increased to 26 significantly upregulated genes, whereas during the 24–35 dph stage, no significantly upregulated phototransduction-related genes were detected. Conversely, the overall number of downregulated genes was minimal, with only 6 significantly downregulated genes detected during the 7–15 dph stage, while no significantly downregulated phototransduction-related genes were observed during other developmental stages.

### 3.7. Expression Analysis of Phototransduction Genes During Early Developmental Stages in Large Yellow Croaker

**Figure 6:**
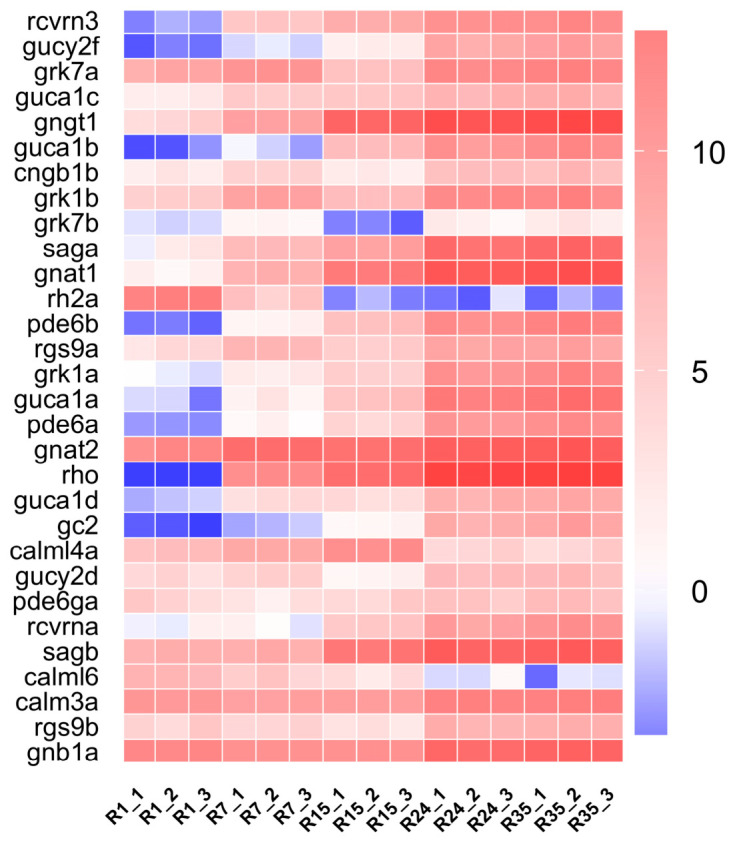
FPKM expression heatmap of phototransduction genes in early developmental stages of *L. crocea*. RNA-seq analysis was performed using three independent biological replicates for each developmental stage (*n* = 3). Expression values were normalized as log_2_(FPKM + 0.1). In the heatmap, white indicates no expression (0), red indicates high expression, and blue indicates low expression. The x-axis represents experimental groups and biological replicates, and the y-axis represents gene names.

Based on functional module classification, phototransduction genes displayed distinct stage-dependent expression patterns during early development (Figure 6). In the photon capture and activation module, *rh2a* was highly expressed at 1 dph and declined thereafter, whereas *rho* remained low at 1–7 dph and was strongly upregulated after 15 dph. Genes in the signal amplification module (*gnat1*, *gnat2*, *pde6a*, *pde6b*, *pde6ga*, *gngt1*, and *gnb1a*) showed uniformly low expression at 1 dph, followed by progressive upregulation, peaking around 24 dph. The ion flux and electrophysiological response module exhibited overall gradual increases across development, with *cngb1b* showing a transient decrease at 7–15 dph. In the termination and recovery module, *grk1a*, *grk1b*, *grk7a*, and *rgs9a* displayed early elevation, transient reduction, and subsequent re-upregulation, whereas *saga*, *sagb*, *rcvrna*, and *rcvrn3* increased continuously from 1 to 35 dph.

### 3.8. Validation of RNA-Seq Results by RT-qPCR

**Figure 7:**
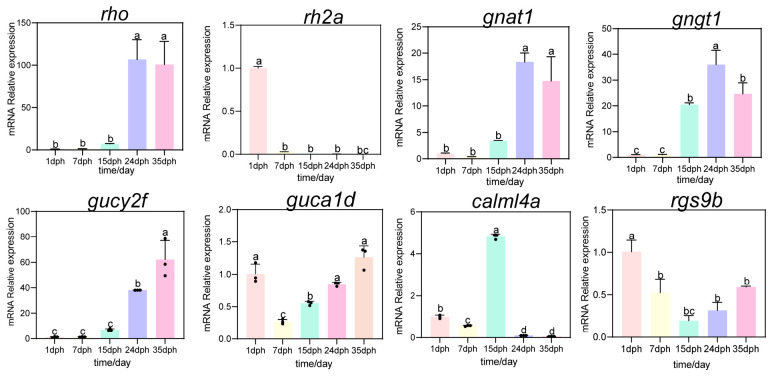
Validation of RNA-Seq results using qPCR. RT-qPCR validation was performed using the same RNA samples as those used for RNA-seq analysis. Three independent biological replicates were analyzed (*n* = 3). Different lowercase letters indicate statistically significant differences among groups (*p* < 0.05).

The reliability of the RNA-seq results was validated by RT-qPCR. Eight genes (*gucy2f*, *guca1d*, *calml4a*, *rgs9b*, *rho*, *rh2a*, *gnat1*, and *gngt1*) were selected from the RNA-seq dataset to verify their expression patterns. RT-qPCR analysis showed expression trends that were consistent with the RNA-seq results. Furthermore, Spearman correlation analysis revealed a significant positive correlation between the two methods (ρ = 0.615, *p* < 0.001), providing quantitative support for the reliability and consistency of the transcriptome sequencing data (Figure 7).

### 3.9. Spectral-Specific Regulation of Visual Development-Related Gene Expression

**Figure 8:**
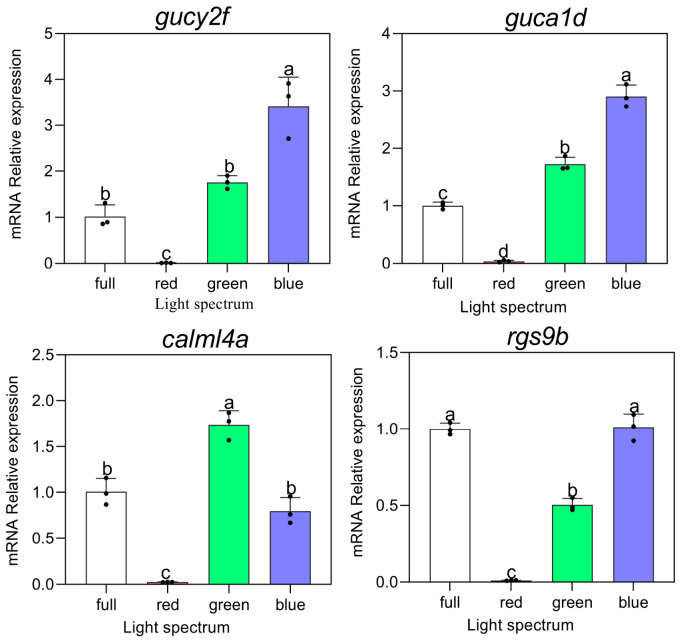
Relative mRNA expression levels of vision-related genes under different light spectra. Data are presented as mean ± SD from three independent biological replicates (*n* = 3). Different lowercase letters indicate statistically significant differences among groups (*p* < 0.05).

The expression levels of vision-related genes were compared among different light spectrum treatments at 15 dph. The expression of phototransduction pathway-related genes (*gucy2f*, *guca1d*, *calml4a*, and *rgs9b*) showed distinct expression patterns among the four light treatments (Figure 8). The expression levels of all four genes were lowest under the red-light treatment (*p* < 0.05). Under blue light, *gucy2f* and *guca1d* reached their peak expression, significantly higher than in other groups. Conversely, *calml4a* showed the highest expression under the green light treatment, while showing no significant difference between the full and blue light conditions. For *rgs9b*, maximal expression was observed under both full and blue light with no statistical difference between them, whereas expression under the green light treatment was significantly lower.

## 4. Discussion

Phototransduction is fundamental to vertebrate visual development. Although phototransduction genes are generally conserved, differences exist among species and developmental stages [27]. In this study, we systematically characterized the composition, structural features, and developmental expression patterns of phototransduction-related genes in large yellow croaker, providing a basis for understanding early visual development in this species.

Based on full-length transcriptome analysis, 30 phototransduction-related genes were identified in the large yellow croaker. These genes covered the major functional modules of the phototransduction cascade, including light perception, signal amplification, and signal termination and recovery, indicating that the large yellow croaker possesses a structurally complete visual signal transduction system [28,29,30,31,32]. Protein domain and subcellular localization analyses showed that these proteins retained highly conserved functional characteristics. Opsins were mainly involved in light perception and signal initiation [33,34], while G proteins and PDE6 mediated signal amplification [35,36], and guanylate cyclases, GRKs, arrestins, and calcium-regulated proteins participated in photoresponse termination and recovery [37,38]. This modular organization was consistent with findings in other teleosts, suggesting strong evolutionary conservation of the phototransduction system [39,40,41,42].

Transcriptomic analysis revealed clear stage-specific expression patterns during early development. Most phototransduction genes showed low expression during 1–7 dph, increased expression during 7–24 dph, and relatively stable expression during 24–35 dph. Only a few genes were upregulated during 1–7 dph, indicating that the visual system was still at an early developmental stage, consistent with previous studies in teleosts. Similar developmental trajectories have been reported in ray-finned fishes, in which phototransduction- and opsin-related genes are progressively activated during retinal differentiation and photoreceptor maturation [43,44,45,46,47]. However, the marked transcriptional activation observed between 15 and 24 dph in large yellow croaker suggests that this developmental window may represent a species-specific period of rapid visual system maturation, consistent with recent histological and transcriptomic observations in this species [48,49]. Notably, the number of upregulated genes peaked during 15–24 dph, and most genes exhibited coordinated expression, suggesting that this period may represent a critical stage for visual system maturation [50]. By 24–35 dph, gene expression became relatively stable, indicating that the major molecular framework of the visual system had largely been established. Since this study did not investigate juvenile or adult stages, further studies are needed to fully clarify the maturation of visual signal transduction in the large yellow croaker.

To determine whether environmental light regulates visual development during critical developmental stages, different spectral treatments were conducted at 15 dph. Phototransduction-related genes exhibited clear wavelength-dependent expression patterns, suggesting that specific light spectra may influence early visual development. Among these genes, *gucy2f* and *guca1d* are involved in cGMP metabolism, while *calml4a* participates in calcium regulation, and *rgs9b* accelerates G protein inactivation to improve temporal resolution [37,38]. These genes represent key downstream components of the phototransduction pathway. Combined with our previous findings on opsins and G proteins, our results indicate that blue and green light were associated with relatively higher expression of phototransduction-related genes under the present experimental conditions, whereas red light was associated with lower expression levels. Furthermore, our previous study demonstrated that blue light preferentially increased the expression of phototransduction-related genes (e.g., *pde6a*) together with genes involved in retinoid metabolism (e.g., *rdh8a* and *adh5*), whereas white light preferentially promoted *rho* expression [50]. These findings suggest that short-wavelength light may coordinately regulate phototransduction together with genes associated with retinoid metabolism during retinal development, thereby facilitating the establishment of the early visual system.

Similar spectral plasticity of visual genes has been reported in teleost fishes, where changes in the ambient light environment modulate the expression of phototransduction- and opsin-related genes. However, the specific response patterns observed in large yellow croaker may reflect adaptations to its coastal marine habitat and early developmental ecology [51]. Notably, the coordinated expression of both upstream photoreceptor genes and downstream phototransduction components suggests that these genes may not be regulated independently but instead may be controlled by a common transcriptional regulatory network. The highly coordinated transcriptional responses observed during both natural development and spectral stimulation further support the possibility that developmental cues and environmental light signals converge on shared upstream regulatory pathways, thereby orchestrating the coordinated expression of phototransduction-related genes. Although this hypothesis requires further experimental validation, it provides a potential framework for understanding the coordinated regulation of visual system development in the large yellow croaker.

The relatively weak effect of red light may reflect the adaptation of large yellow croaker to turbid coastal waters, where long-wavelength light attenuates rapidly [52]. However, this hypothesis still requires further validation. Although blue and green light significantly regulated phototransduction-related genes, these molecular responses may not necessarily correspond to improved visual function. Previous studies showed that loss of short-wavelength-sensitive genes in some teleosts did not markedly impair visual adaptation [6], while excessive blue light may induce retinal damage in mammals [24]. Therefore, the transcriptional responses observed in the present study should be interpreted as molecular indicators of visual system development rather than direct evidence of enhanced visual performance. Whether the spectral responses observed here enhance visual function remains unclear and requires electrophysiological, behavioral, and long-term exposure studies. In addition, irradiance power and photon flux density were not standardized among spectral treatments, and the observed differences may therefore reflect combined effects of spectral composition and photon input.

Overall, this study revealed the developmental and environmental regulation of the early visual system in large yellow croaker and demonstrated that the overall organization and developmental dynamics of the phototransduction pathway are largely conserved among teleosts, while the timing of transcriptional activation and spectral responsiveness exhibit characteristics that may be specific to large yellow croaker. These findings provide new insights into visual development and light adaptation in teleosts. Nevertheless, several limitations should be acknowledged. The spectral experiment used continuous white light as the reference treatment to simulate current hatchery practices but did not include a dark-control group, which limits further mechanistic interpretation of light-dependent gene regulation. Moreover, because irradiance and photon flux density were not standardized among the different LED treatments, the effects of wavelength and photon flux were confounded and therefore cannot be statistically separated in the present experimental design. In addition, although three biological replicates per group are commonly used in gene expression studies, the relatively small sample size limits the statistical power of the analyses. Future studies incorporating larger sample sizes, additional light-control treatments, and functional approaches will help clarify the upstream regulatory mechanisms governing phototransduction gene expression during retinal development in the large yellow croaker.

## 5. Conclusions

This study systematically identified and characterized phototransduction-related genes in large yellow croaker and revealed their dynamic expression patterns during early development as well as their responses to different light spectra. To our knowledge, this is the first systematic characterization of the phototransduction pathway in *L. crocea*, providing a comprehensive framework linking gene composition, developmental expression dynamics, and environmental light responses. The results demonstrated that the phototransduction system is highly conserved and undergoes rapid functional development during the critical developmental window of 15–24 dph. Our findings further suggest that phototransduction-related genes are coordinately regulated during retinal development and exhibit distinct spectral responsiveness under different light environments. These findings advance current understanding of the molecular basis of visual system development in marine teleosts, provide a molecular basis for understanding early visual development and light adaptation in large yellow croaker, and offer theoretical support for optimizing light conditions in aquaculture.

## Data Availability

The raw sequence data reported in this paper have been deposited in the Genome Sequence Archive in the National Genomics Data Center, China National Center for Bioinformation/Beijing Institute of Genomics, Chinese Academy of Sciences (GSA: CRA035056), which are publicly accessible at https://ngdc.cncb.ac.cn/gsa (accessed on 15 January 2026).

## Supplementary Materials

The following supporting information can be downloaded at: https://www.mdpi.com/article/10.3390/genes17070788/s1.
