# Supplementary material for: Developmental and Light-Induced Expression Patterns of Phototransduction Pathway Genes in Large Yellow Croaker (Larimichthys crocea)
Source: Genes (Basel). 2026 Jul 10;17(7):788. doi: 10.3390/genes17070788 (PMC13408731; doi:10.3390/genes17070788)
Supplement: Supplementary file 1 [file genes-17-00788-s001.zip › genes-4373701-supplementary.pdf]

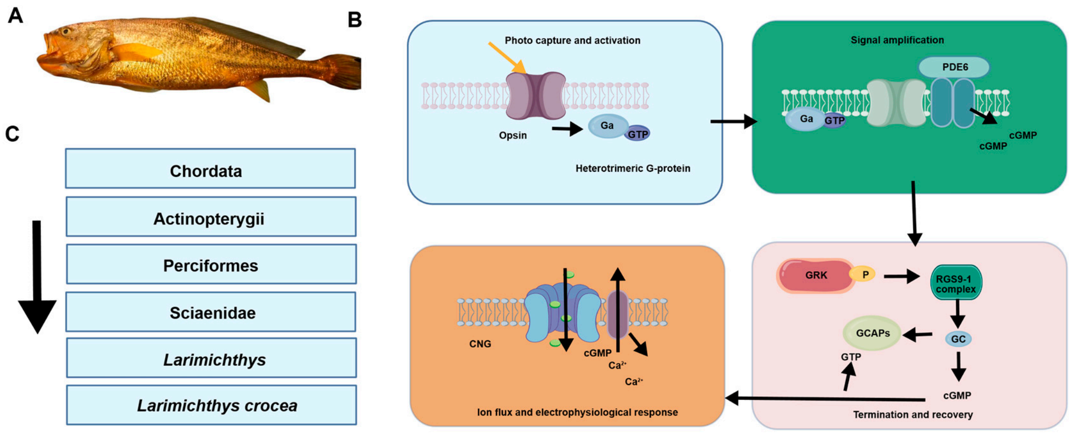

Figure S1. Visual system, phototransduction pathway, and phylogenetic position of the large yellow croaker (*Larimichthys crocea*). (**A**) Adult morphology of the large yellow croaker. (**B**) Schematic overview of the vertebrate phototransduction cascade, organized into four functional modules: photon capture and activation, signal amplification, ion flux and electrophysiological response, and termination and recovery. (**C**) Schematic illustration of the phylogenetic position of *L. crocea* within teleost fishes.

Table S1. Amino acid sequences used for phylogenetic tree construction.

Gene	Species	Accession number
Lc-rcvrn3	*Larimichthys crocea*	XP_010736010.1
Lc-gucy2f	*Larimichthys crocea*	XP_010755434.3
Lc-grk7a	*Larimichthys crocea*	XP_010731605.1
Lc-guca1c	*Larimichthys crocea*	XP_010741661.1
Lc-gngt1	*Larimichthys crocea*	XP_010739845.1
Lc-guca1b	*Larimichthys crocea*	XP_010745639.1
Lc-cngb1b	*Larimichthys crocea*	XP_019135117.2
Lc-grk1b	*Larimichthys crocea*	XP_019124991.1
Lc-grk7b	*Larimichthys crocea*	XP_010735741.3
Lc-saga	*Larimichthys crocea*	XP_027143325.1
Lc-gnat1	*Larimichthys crocea*	XP_010731081.1
Lc-rh2a	*Larimichthys crocea*	XP_019118059.2
Lc-pde6b	*Larimichthys crocea*	XP_019115676.1
Lc-rgs9a	*Larimichthys crocea*	XP_019123785.1
Lc-grk1a	*Larimichthys crocea*	XP_027146663.1
Lc-guca1a	*Larimichthys crocea*	XP_010737222.1
Lc-pde6a	*Larimichthys crocea*	XP_010748161.3
Lc-gnat2	*Larimichthys crocea*	XP_010739101.1
Lc-rho	*Larimichthys crocea*	XP_010740392.1
Lc -guca1d	*Larimichthys crocea*	XP_010738243.1
Lc-gc2	*Larimichthys crocea*	XP_019119511.2
Lc-calml4a	*Larimichthys crocea*	XP_010731048.1
Lc-gucy2d	*Larimichthys crocea*	XP_027140340.1
Lc-pde6ga	*Larimichthys crocea*	XP_019116624.1
Lc-rcvrna	*Larimichthys crocea*	XP_010747843.3
Lc-sagb	*Larimichthys crocea*	XP_010748088.1
Lc-calml6	*Larimichthys crocea*	XP_019118496.1
Lc-calm3a	*Larimichthys crocea*	XP_010742180.1
Lc-rgs9b	*Larimichthys crocea*	XP_010734786.1
Lc-gnb1a	*Larimichthys crocea*	XP_010732274.1

Table S2. Primers used in qRT-PCR for large yellow croake gene analysis.

Gene	Primer	Sequence of primer (5′–3′)	Accession numbers
β-actin	*β-actin*-F	TCGTGCGTGACATCAAGGAGAA	XM_010733633.3
	β-actin-R	ACCGAGGAAGGATGGCTGGAA	
18SrRNA	*18SrRNA*-F	CCGGGGCCATGATTAAGAGG	JN211788.1
	18SrRNA-R	CGCCGGTCCAAGAATTTCAC	
gucy2f	*gucy2f*-F	AGGTCTGACTATGCCTCGGT	XM_010757132.3
	gucy2f-R	GCAGCCCAGTGGATTCCATA	
guca1d	*guca1d*-F	ATCGACGTGAAAGGAGACGG	XM_010739941.3
	guca1d-R	ACACTGGCGTTAGGTCCATC	
calml4a	*calml4a*-F	GATACATCCAGGCCTCTGAGC	XM_010732746.3
	calml4a-R	TGACAACCCCGTTTGACTTGA	
rgs9b	*rgs9b*-F	TCAAACCAGATGCGTCCCTC	XM_010736484.3
	rgs9b-R	CGTATCTTCAACCGGCCACT	

Table S3. Physicochemical properties of phototransduction-related proteins in *Larimichthys crocea.*

Gene	Number of amino acids	Molecular weight (Da)	pI	Subcellular location	Alpha helix/ %	Beta turn/ %	Random coil/ %	Extended strand/ %
calm3a	149	16837.59	4.09	Cell membrane, Cytoplasm	60.4	0	30.87	8.72
calml4a	153	17775.74	7.7	Cell membrane, Cytoplasm, Nucleus	60.13	0	31.37	8.5
calml6	166	18816.42	4.62	Nucleus	61.45	0	31.33	7.23
cngb1b	899	100657.84	8.95	Cytoplasm	36.82	0	54.95	8.23
gc2	1150	129172.53	7.9	Cell membrane, Cytoplasm	33.91	0	49.74	16.35
gnat1	350	40049.86	5.43	Cell membrane, Cytoplasm, Golgi apparatus, Nucleus	53.14	0	34.86	12
gnat2	350	39669.39	5.21	Cell membrane, Cytoplasm, Golgi apparatus, Nucleus	53.14	0	35.14	11.71
gnb1a	340	37330	5.6	Cell membrane	8.24	0	48.53	43.24
gngt1	73	8297.68	4.69	Cytoplasm, Nucleus	50.68	0	41.1	8.22
grk1a	561	64194.57	5.85	Cytoplasm, Nucleus	42.07	0	47.24	10.7
grk1b	559	63672.03	7.5	Cytoplasm, Nucleus	37.92	0	51.34	10.73
grk7a	558	63604.14	8.45	Cytoplasm, Nucleus	41.22	0	49.28	9.5
grk7b	552	63901.34	8.81	Cytoplasm	42.03	0	47.64	10.33
guca1a	189	21851.88	4.96	Cell membrane	56.61	0	37.57	5.82
guca1b	197	22999.08	4.66	Cell membrane	57.36	0	38.07	4.57
guca1c	189	21866.98	4.31	Cell membrane	59.79	0	35.45	4.76
guca1d	185	21384.28	4.36	Cell membrane	54.59	0	39.46	5.95
gucy2d	238	27214.72	5.55	Extracellular, Mitochondrion, Nucleus	47.06	0	50.42	2.52
gucy2f	1107	124746.62	7.05	Cell membrane, Cytoplasm	36.04	0	47.06	16.89
pde6a	857	99351.07	5.5	Cytoplasm	48.89	0	42.47	8.63
pde6b	856	98693.38	5.45	Cytoplasm	50.82	0	42.06	7.13
pde6ga	104	11441.19	8.64	Nucleus	12.5	0	24.04	63.46
rcvrn3	190	21950.76	5.27	Cell membrane, Cytoplasm	55.79	0	39.47	4.74
rcvrna	203	23394.3	5.08	Cell membrane, Cytoplasm	53.2	0	41.38	5.42
rgs9a	481	56134.77	9.53	Cytoplasm	51.35	0	41.58	7.07

rgs9b	657	74997.92	9.14	Cytoplasm	36.53	0	57.38	6.09
saga	410	45672.39	5.81	Cytoplasm	12.68	0	62.68	24.63
sagb	386	43629.1	6.34	Cytoplasm	17.62	0	55.96	26.42

Table S4 Functional Categorization of 30 Phototransduction Genes in *L. crocea*

Gene	Molecular Function (MF)		Functional Module
rho<br>*rh2a*	GO:0008020	G-protein coupled photoreceptor activity	Photon Capture and Activation Module
gnat1	GO:0031683	G-protein beta/gamma-subunit complex binding	
gnat2	GO:0031683	G-protein beta/gamma-subunit complex binding	
pde6a<br>*pde6b*	GO:0004114	3',5'-cyclic-nucleotide phosphodiesterase activity	Module Signal Amplification Module
pde6ga	GO:0047555	3',5'-cyclic-GMP phosphodiesterase activity	
gngt1	GO:0031681	G-protein beta-subunit binding	
gnb1a	GO:0031682	G-protein gamma-subunit binding	
cngb1b	GO:0030553	cGMP binding	
calm3a<br>*calml4a*<br>*calml6*<br>*guca1a*<br>*guca1b*<br>*guca1c*<br>*guca1d*	GO:0005509	calcium ion binding	Ion Flux and Electrophysiological Response
gucy2d<br>*gucy2f*<br>*gc2*	GO:0004383	guanylate cyclase activity	
grk1a<br>*grk7b*<br>*grk7a*<br>*grk1b*	GO:0004703	G-protein coupled receptor kinase activity	Termination and Recovery Module
sagb<br>*saga*	GO:0001664	G-protein coupled receptor binding	
rgs9a<br>*rcvrna*	GO:0005509	calcium ion binding	
rcvrn3<br>*rgs9b*	GO:0005096	GTPase activator activity	
